# Nasogastric Tube Feeding-Induced Esophageal Bezoar: Case Description

**DOI:** 10.1155/2017/1365736

**Published:** 2017-04-05

**Authors:** Jad A. Degheili, Mikhael G. Sebaaly, Ali H. Hallal

**Affiliations:** ^1^Division of General Surgery, Department of Surgery, American University of Beirut Medical Center, Riad El-Solh, Beirut 1107 2020, Lebanon; ^2^Department of Diagnostic Radiology, American University of Beirut Medical Center, Riad El-Solh, Beirut 1107 2020, Lebanon

## Abstract

*Background*. Bezoars are well established entities causing gastrointestinal obstructions. Depending on the prominent constituent of these bezoars, the latter are divided into four subtypes: pharmacobezoars, lactobezoars, trichobezoars, and phytobezoars. Less frequently reported types of bezoars are reported including those formed secondary to nasogastric tube feeding with casein-based formulas.* Case Presentation.* A 69-year-old male presented following cardiac arrest postmyocardial infarction. Patient sustained anoxic brain injury after resuscitation, rendering him ventilator dependant along with nasogastric tube feeding, initially. Dislodging of the nasogastric tube at one time rendered it difficult to reinsert it, with investigation showing the presence of calcified material within the distal oesophagus, mainly composed of casein-based products secondary to enteral feeding.* Conclusion.* Bezoars are well known to cause gastrointestinal obstructions due to their indigestible characteristics within the alimentary tract. More rare causes of bezoars include those formed from casein-based feeding formulas administered to patients with sustained enteral feeding. Esophageal obstruction, secondary to casein-based bezoars, occurs due to multiple risk factors, especially in those necessitating intensive care. Approach in such scenarios requires a multiteam approach.

## 1. Background

Gastrointestinal bezoars are indigestible conglomerations, causing various degree of obstruction within the alimentary tract. Depending on the prominent constituent of the conglomerate, gastrointestinal bezoars can be classified accordingly. Pharmacobezoars (medication-induced), lactobezoars (neonates fed improper formula), trichobezoars (ingested hairs), and phytobezoars (undigested food materials) are among the commonly reported forms of bezoars.

Phytobezoars, which are the most common type, constitute mainly fibers, skin, and seeds of fruits and vegetables; in reference to the high amount of cellulose, hemicellulose, lignin, and tannins these elements contain, this renders them indigestible. In Japan, persimmon is a common constituent of phytobezoars, as it is highly consumed in the Japanese culture. Trichobezoars, formed from hair balls trapped within the GI system, are almost always diagnosed in young females, possessing trichotillomania and trichophagia. Despite the stomach being the most common location, bezoars can also be present primarily within the small bowels or migrate there from the stomach. Rapunzel syndrome [1] represents hair balls extending from the stomach to the small and large bowels, respectively.

Extended-release pills or those with enteric coatings made of cellulose acetate are not digested and form pharmacobezoars. Other drugs such as cholestyramine, aluminium-containing antacids, sucralfate, and aspirin can also form pharmacobezoars [2]. Lactobezoars, which mainly affect milk-fed infants and toddlers, are mainly composed of milk and mucus. To sum up, all indigestible food materials and foreign bodies are nidus for bezoars formation.

A less common and rarely identified form of bezoar is that of feeding formulas administered to those with a feeding tube, which are unable to meet their nutritional needs orally, such as those set on mechanical ventilators. Rarely, such bezoars are present within the oesophagus, as in our case.

Nasogastric tube feeding-induced bezoars are less frequently reported. We hereby present a case of esophageal bezoar secondary to administration of casein-based feeding formulas in an intensive care patient.

## 2. Case Presentation

A 69-year-old man, with multiple medical comorbidities, including coronary artery disease, presented to our emergency department, following cardiac arrest, postmyocardial infarction. After successful resuscitation, patient sustained a permanent anoxic brain injury, rendering him dependant on a mechanical ventilator and nasogastric (NG) tube feeding.

Accidental dislodging of the NG tube during patient routine repositioning renders it difficult to reinsert it again. Resistance at a distance of around 40 cm from the nostrils, that is, distal third of the oesophagus, was encountered.

**Figure 1:**
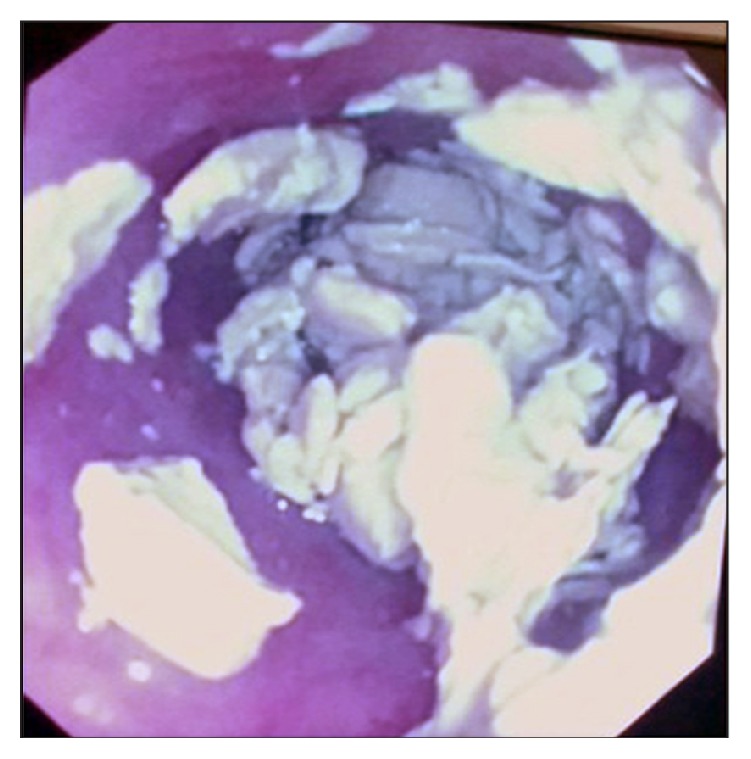
Endoscopic view of the upper gastrointestinal tract, revealing the presence of calcified debris, within the esophagus, secondary to nasogastric tube feeding.

Both a tracheostomy and a gastrostomy tube insertion were advised. A prior upper GI tract endoscopy revealed the presence of calcified material, most likely representing an esophageal bezoar (Figure 1).

**Figure 2:**
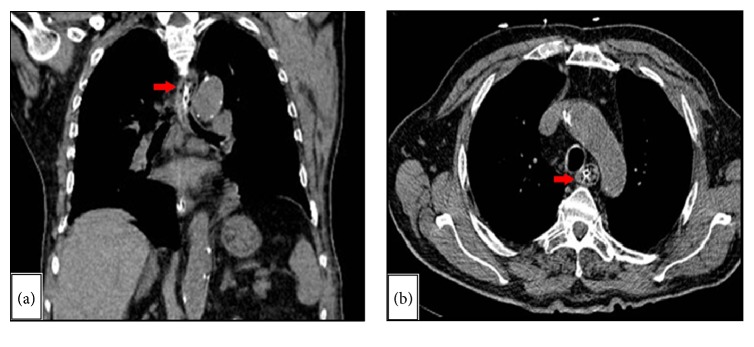
Computed tomography of the chest and upper abdomen ((a) coronal; (b) axial) with minimal administration of contrast through an NG tube, revealing the extent of the bezoar (red arrow) within the upper third of the esophagus.

The esophageal extent of this bezoar was then assessed by means of a computed tomography of the chest and upper abdomen, with minimal contrast administered through the NG tube (Figures 2(a) and 2(b)). Due to patient's terminal condition, feeding was instead initiated by securing a surgically placed jejunostomy feeding tube.

## 3. Discussion

Feeding-formulas bezoars are mostly casein-based. Proteins in these feeding formulas can be divided into either casein or whey. The two exhibit variable amino acid compositions. As such, they differ in their gastric emptying, absorption, and serum kinetics. Cow's milk, which is the major constituent of these feeding formulas, is casein-based rather than whey-based, as in human milk. Cow's milk contains 80% casein-protein versus 20% whey-protein. Rarely reported, casein-based products can form nidus for esophageal bezoars [3].

Bezoars of the GI system exhibit variable incidences among studies. In general, gastric bezoars are present in 0.5% of cases undergoing esophagogastroduodenoscopy (EGD). Nevertheless, this figure is variable among studies. Mihai et al. noted an incidence of 0.068% of gastric bezoars among all endoscopies performed over a two-decade period [4]. On the other hand, Kadian et al. and Ahn et al. reported a similar incidence of 0.43% of all gastroscopies performed, over a 4-year and 7-year period, respectively [5, 6]. Intestinal bezoars constitute 0.4 to 4.8% of all cases presented as small bowel obstruction. Such reported incidences are variable as they do reflect the study population food culture.

Several risk factors can predispose to the formation of gastrointestinal bezoars secondary to delayed gastric emptying, gastric stasis and reduce in gastric acidity. Previous gastric surgeries including partial gastrectomy, vagotomy, and pyloroplasty, peptic ulcer disease, achalasia, GI malignancies, Crohn's disease, hypothyroidism, presence of hiatal hernia, and in elderly patients neuropathic or myotonic dystrophy are among the major ones.

As previously stated, esophageal bezoars are rarely reported. The medical literature has described several cases of esophageal obstruction due to bezoars with no previous warnings. García-Luna et al. reported three cases of esophageal bezoars due to feeding by nasogastric tube and coadministration of sucralfate [7]. On the other hand, Marcus et al. reported a similar case where esophageal obstruction occurred with casein-containing formulas [8]. Caldeira et al. conducted a three-year retrospective study on 1367 intensive care unit patients [9]. Of those, 1003 had enteral feeding and 9 (0.9%) only developed esophageal impaction or esophageal bezoar formation. In all nine cases, esophageal bezoar was secondary to solidification of casein-based formulas within the oesophagus. In spite of the incidence being low, its impact is much greater. Those with esophageal bezoar witnessed a delay in ICU stay equivalent to twice that of other patients in the same ICU setting.

NG tubes that were incorrectly placed or displaced within the distal oesophagus [2] are risk factors for esophageal bezoars. Marcus et al. [8] had postulated that casein-based enteral feedings, such as Ensure, could conglomerate in the presence of an acidic environment having a pH of less than 5, especially that they are deficient in enzymolytic substances such as pepsin and pancreatic juices. Such reflux of gastric secretions occurs in patients who are sustained on mechanical ventilators, in supine position, and those on sedative drugs which hamper overall gastrointestinal motility. Turner et al. [10] have also confirmed this postulate in in vitro experiments. They had proven that casein-containing formulas solidify within a 5-minute period, in acidic media, having a pH value less than 5, hence the risk of bezoar formation in patients with gastroesophageal reflux disease. Confirmation of the proper placement of the nasogastric feeding tube within the stomach, under radiography, is crucial and the standard of care [11]. This will avoid a rare but serious and deadly complication of esophageal perforation from misplaced NG tubes [12].

In return, gastroesophageal reflux can be exacerbated by supine positioning of the patient [13], as it will induce relaxation of the lower esophageal sphincter. Wang et al. [14] demonstrated that 40% of patients with type II diabetes mellitus showed symptoms of reflux, as well. Hampel et al. [15] also showed that obesity and overweight are risk factors for gastroesophageal reflux. Others risk factors include hypothyroidism and neurological disease such as Guillain-Barre syndrome, myasthenia gravis, head injury, and other brain insults.

Studying the composition of those esophageal bezoars, Tawfic et al. reported a constituent made of a mixture of amorphous materials formed from bacteria, foci of calcifications, and food particles, along with squamous hyperplasia [2].

Several radiological modalities have been utilised for visualization of gastrointestinal bezoars. Ripollés et al. [16] compared the three most commonly used modalities: abdominal radiography (KUB), ultrasonography, and computed tomography, respectively. On KUB, bezoars are suspected when mottled radiotransparencies in the interstices of solid matter are identified. Signs of small bowel obstructions may or may not be evident on such radiological imaging. Nevertheless, KUB images are not specific for bezoars as they can be easily mistaken with an abscess or presence of feces within the colon. In their series, Ripollés et al. and Verstanding et al. reported an 18% and 10% rate of accurate identification of bezoars on KUB alone, respectively [16, 17]. Sonographic confirmation of bezoar is established by the detection of an intraluminal mass with a hyperechoic arc-like surface and a marked acoustic shadow. On CT imaging, a bezoar appears as a low-density intraluminal mass containing air-bubbles with a characteristic mottled appearance. The bowel loops proximal to the mass appear dilated, while the distal loop appeared normal or collapsed. This study represents the first ever published data on the usefulness of each imaging modality and comparison among the three of them. The authors summed up that CT scan is the most useful modality in diagnosing bezoars than either conventional KUB or ultrasound. The computed tomography images have added further characteristics and then the other modalities, including the determination of the exact point of obstruction, revealing whether the bezoar is the primary cause of the obstruction and not any other pathology, along with the detection of any other additional gastric or intestinal bezoars. This is crucial as Ripollés et al. found in their series a 53% chance of concomitant gastric bezoars in patients with intestinal bezoars [16]. Others have reported a concurrent incidence of gastric bezoars of 17–21% and the rate of recurrence of 13.8% [12]. As such, bezoars located elsewhere within the gastrointestinal tract must be ruled out in any patient with an established bezoar [16].

In our present case, several risk factors were identified including feeding in a supine position, mechanical ventilation, anoxic brain injury, nasogastric tube feeding, and the use of casein-containing formulas.

Although as previously stated upper gastrointestinal bezoars can be diagnosed radiologically, endoscopy is considered the best diagnostic and therapeutic tool. Through endoscopy, bezoars can be removed using endoscopic snares or forceps, dissoluted, and fragmented using different lytic enzymes such as papain, which is extracted from the Carica Papaya plant, N-acetylcysteine, or cellulose or using several effervescent solutions such as sodium bicarbonate and Coca-Cola™ [18, 19].

Several maneuvers could be performed to circumvent such complications. These include administration of proton pump inhibitors, feeding in a semirecumbent position, administration of non-casein-containing formulas, the use of prokinetics, or simply periodic hydration or adequate flushing through the nasogastric route. Other alternatives include endoscopic introduction of a nasojejunal (NJ) tube instead [9].

In cases where chronic ventilation is expected, percutaneous gastrostomy or jejunostomy can be used to decrease the risk of esophageal reflux and hence bezoar formation.

In conclusion, a rare case of esophageal bezoars causing obstruction has been described along with detailed revision of the possible risk factors, preventions, and treatment approaches.

## 4. Conclusion

Bezoars are well known to cause gastrointestinal obstructions due to their indigestible characteristics within the alimentary tract. Despite the more common types of bezoars including pharmacobezoars, lactobezoars, trichobezoars, and phytobezoars, a more rare cause of bezoars include those formed from casein-based enteral feeding formulas administered to patients on respiratory ventilators. Esophageal obstruction, secondary to casein-based bezoars, occurs due to multiple risk factors. Management approach in such scenarios requires a multiteam approach from the intensivist, endoscopist, and the surgical team, as well.
